# Women’s participation in household decision-making: Qualitative findings from the Shonjibon Trial in rural Bangladesh

**DOI:** 10.1371/journal.pgph.0002907

**Published:** 2024-06-17

**Authors:** Elizabeth K. Kirkwood, Jasmin Khan, Mohammad Mehedi Hasan, Afrin Iqbal, Tazeen Tahsina, Tanvir Huda, John Frederick Hoddinott, Tracey Lea Laba, Sumithra Muthayya, Nicholas Goodwin, Munirul Islam, Emwinyore Agho Kingsley, Shams E. Arifeen, Michael J. Dibley, Neeloy Ashraful Alam

**Affiliations:** 1 Faculty of Medicine and Health, Sydney School of Public Health, The University of Sydney, Sydney, New South Wales, Australia; 2 Maternal and Child Health Division, International Centre for Diarrhoeal Disease Research Bangladesh, Dhaka, Bangladesh; 3 Division of Nutritional Sciences, Cornell University, Ithaca, New York, United States of America; 4 Centre for Health Economics Research and Evaluation, University of Technology Sydney, Sydney, New South Wales, Australia; 5 Clinical and Health Sciences, University of South Australia, Adelaide, Australia; 6 The Sax Institute, Glebe, New South Wales, Australia; 7 Nutrition and Clinical Services Division, International Centre for Diarrhoeal Disease Research Bangladesh, Dhaka, Bangladesh; 8 School of Health Sciences, Western Sydney University, Penrith, New South Wales, Australia; PLOS: Public Library of Science, UNITED STATES

## Abstract

A key element of women’s empowerment is the ability to participate in household decision-making. This study presents the qualitative results from the Shonjibon Cash and Counselling Trial baseline process evaluation with the aim of exploring the status of women’s decision-making at the trial’s outset and to facilitate the exploration of any changes in women’s empowerment over the course of the trial. Between January and March 2021, we conducted forty-one in-depth interviews with pregnant women in rural Bangladesh. The research team translated, transcribed, coded, and discussed the interviews. We used thematic analysis to examine women’s experience and perceptions on household decision-making. The key findings that emerged; women jointly participated in financial decision-making with their husbands; men made the final decision regarding seeking healthcare, and women solely made choices regarding infant and young child feeding. Our findings revealed that women felt that they needed to discuss their plans to go outside the house with their husbands, many perceived a lack of importance in the community towards women’s participation in decision-making. This study documents current contextual information on the status of women’s involvement in household decision-making and intrahousehold power dynamics at the start of the Shonjibon Cash and Counselling Trial.

## Introduction

All women have the right to be empowered, identify their needs, act upon them, and have control over their lives [1,2]. One’s decision-making ability can be used as a gauge to measure agency and is a proxy measure of empowerment [3,4]. A key component of empowerment is the ability to participate in decision-making including but not limited to those involving household purchases, financial decisions, and the ability to move about freely [4,5]. To improve health outcomes for women and children, women need to be able to fully participate in decision-making [6]. In the context of Bangladesh, women’s empowerment, is reflected through decision-making ability, and potentially enabled by initiatives such as mHealth and cash transfers, correlating with improved health outcomes, thereby highlighting the interconnectedness of these elements within the broader socio-economic landscape.

Women’s empowerment, including active participation in decision-making, is positively associated with improved dietary quality and dietary diversity within households yet will vary with individuals across the life course [7,8]. Women’s decision-making ability is positively associated with household nutritional status, reproductive and child health and expenditure on education and health [6,8,9]. However, empowerment did not benefit all household members equally in terms of dietary diversity and nutrient intakes with a gender bias shown toward adolescent boys. [7]. When women are engaged in decision-making, this directly supports greater well-being for society, households and individuals [10].

Cash transfers targeted at women can stimulate women’s economic empowerment and improve agency and decision-making ability [11–15]. Social safety net programs that increase women’s control of such income are also associated with better nutrition outcomes for themselves and their families [16–19]. When women are involved in nutrition-sensitive programmes that use cash transfers, certain aspects of women’s empowerment are enhanced, such as changes in gender roles and intrahousehold bargaining power [12]. There is some evidence on the impact of unconditional cash transfers on women’s empowerment, the study of cash transfer programme in Pakistan found positive impacts on some of the variables that are used to measure women’s empowerment and ability to participate in decision making [20]. However, the evidence on the impact of unconditional cash transfers on women’s empowerment is limited [20].

The influence of women’s empowerment on child nutritional status and health outcomes for women and children is increasingly recognised [21–24]. Evidence shows that when women experience inadequate decision-making power and lack control over resources, it contributes to undernutrition for themselves and their children [21,24,25]. Research from Bangladesh has revealed disempowerment of women is strongly associated with maternal undernutrition and low birth weight babies [26]. A systematic review in South Asia found relative differences in household members’ bargaining power, income, food behaviours, social status, and interpersonal relationships determined their food allocation and reflected social and cultural gender-based discrimination [27]. These studies highlight the need for a more context-specific evaluation of the role of women’s decision-making ability and health outcomes.

The expanse and proliferation of mobile technologies, especially in low resource settings, offers an innovative way to communicate public health messages [28–30]. Mobile health (mHealth) interventions hold the potential to enhance women’s independence in accessing healthcare services and information, thereby bolstering their ability to make informed health-related decisions [31]. Numerous health initiatives using digital technology strive to enhance the health of women in low- and middle-income countries, with a particular focus on maternal and child health [32–35]. Nonetheless, gender-based disparities present a hurdle for women, as they encounter lower literacy rates and reduced access to mobile technology, which can impede the adoption and effectiveness of health interventions delivered through digital platforms [36–38]. Studies have also demonstrated that mHealth interventions can have a positive impact on transforming gender dynamics by enhancing access to healthcare resources, empowering women in decision-making, and facilitating communication between spouses [36].

Women of low socio-economic status in South Asia experience barriers to exercising their ability to have authority over decisions that directly impact their lives [39]. Bangladeshi women face gender-based inequities which impede their ability to participate in the decision-making process. These inequities are further reflected in the disproportionate access of women to education and employment, their limited access to food and health care and their lesser say in household decision-making powers [40–42].

This study aims to explore women’s participation in intra-household decision-making at the baseline of the Shonjibon Cash and Counselling Trial in rural Bangladesh [43]. This paper documents a section of the baseline process evaluation findings from the start of the SCC Trial. Our objective is to understand the women’s perceptions, attitude and practice regarding decision-making related to finance, seeking health care, freedom of movement and the overall importance of women’s participation in decision making and in order to assess changes in women’s empowerment due to their participation in the study.

### The Shonjibon Cash and Counselling Trial

The Shonjibon Cash and Counselling (SCC) Trial is a longitudinal cluster randomised controlled trial evaluating the impact of unconditional cash transfers with nutrition counselling on pregnant women’s birth outcomes in rural Bangladesh [43] (Shonjibon stands for Shustho Notun Jibon in Bangla; translated, it means ’healthy new life’). The SCC Trial plans to improve nutrition knowledge and influence maternal and child nutrition practices through behaviour change communication (BCC).

Women in both arms of the SCC trial receive a mobile phone. The intervention arm receives (1) unconditional cash transfer of 1000 Bangladeshi Taka (approximately US$12.50) received monthly via BKash mobile banking app. (2) nutrition BCC delivered on a specially tailored app on a smartphone (audio, video, and animation); and (3) direct nutrition counselling from a call centre. The mobile app is called "Soi Barta", which translates from Bangla to mean "message from a friend". The BCC entails women downloading the “Soi Barta” app with help from the study team and throughout their pregnancy and birth will receive gestational age specific nutrition and livelihood focused weekly messages or messages tailored to the age of their child.

The SCC Trial recruited pregnant women (2840 mother-child dyads) and will follow up with up over 24 months, from recruitment in early pregnancy until the child is 18 months of age. Women in the control arm receive a mobile phone and the current government of Bangladesh health and nutrition services. The field staff and research team had no prior relationship with the participants of the study nor any knowledge of the research teams personal goals for undertaking this research.

The study setting is in Sirajganj, a district in northern Bangladesh, one of the poorest regions in the country, according to the World Food Programme Poverty Maps [44]. Both quantitative and qualitative approaches were employed to address the study objectives. Separate protocol papers describe the design details of the SCC Trial [43,45]. It is imperative to assess if an intervention, such as the SCC Trial, can influence changes in women’s agency and decision-making ability. A recent report from The United Nations showed that only around half of the married or partnered women globally can freely make their own decisions about health care, contraceptive use or sexual relations [46]. Gender inequalities often disadvantage health outcomes for women due to the lack of decision-making autonomy [47].

## Methods

### Ethics statement

The Ethical Review Committee (ERC) of the icddr,b, and the Human Research Ethics Committee (HREC) of University of Sydney have granted ethics approval for this study. Written or oral informed consent was obtained from all participants prior to enrolment in the study. Women were informed they could leave the study at any time without any penalty. We have registered the trial in the Australian New Zealand Clinical Trials Registry (ACTRN12618001975280).

This qualitative study explored the experiences of women in the SCC Trial’s participation in decision-making. This study is part of the assessment of the impact of the SCC trial on women’s empowerment and the details of this component can be found in a separate protocol paper [45]. Married women aged between 15–49 years who were permanent residents of the study area and had a positive pregnancy test (gestational age under 90 days) area were enrolled [43]. Women that participated in the study gave oral informed consent. We obtained consent from those participants under the age of 18 directly as those in this age group are considered adults in Bangladesh, and we therefore did not obtain consent from parent or legal guardian, as approved by ethics committees both at icddr,b and the University of Sydney. Additional information regarding the ethical, cultural, and scientific considerations specific to inclusivity in global research is included in Human Subjects Research Checklist (Appendix A).

As part of the baseline process evaluation, we conducted forty-one in-depth interviews with pregnant women between January and March 2021. The implementation team provided a list of potential households from the project. Researchers approached households and invited them to be part of this study. Women were purposively selected and interviewed approximately two months after the start of the intervention. None of the women asked to participate refused to take part in the study. The researchers purposively selected interviewees from Ullapara and Kamarkhanda upazillas (sub-districts) located in Sirajganj, Bangladesh, between January and March 2021. The field team from the International Center for Diarrhoeal Disease Research, Bangladesh (icddr,b) conducted the interviews, and consisted of an experienced anthropologist, along with three local (female) researchers with interest and experience in women’s and children’s health.

Interview guidelines were pilot tested prior to the interviews taking place. Each interview lasted between forty-five minutes to one and a half hours and were conducted within the home. Interview notes were made during and after the interview. No repeat interviews were conducted. Transcripts were not returned to participants for comments or corrections. Data saturation was discussed within the research team and the field team. The field team reported hearing repeated information on the key themes necessary to answer our research question.

### Data analysis

We utilized both inductive and deductive coding approaches in our study. We employed both a priori and open coding in our data analysis. First, we developed a set of a prior code based on the interview guidelines. EKK undertook initial coding using the draft a prior code list and discussed coding details with Bangla-speaking NAA and J.K. We applied an open coding approach to include any newly relevant data in accordance with our research question. Thematic analysis was used to examine the participants’ experience of decision making [48]. We recorded interviews using digital audio recording devices. The research team transcribed interviews verbatim in Bangla at icddr,b and entered data into Microsoft Word files. A researcher from icddr,b translated the transcripts into English. Quality control was maintained by NAA (senior health social scientist), who checked the transcripts at random intervals to ensure the accuracy of transcriptions. Participants did not provide feedback on the findings. NVivo software (V12) was used to organise, code, categorise and compile the data [49]. We have attached the Consolidated Criteria for Reporting Qualitative Research (COREQ) S2 Checklist [50].

## Results

**Fig 1:**
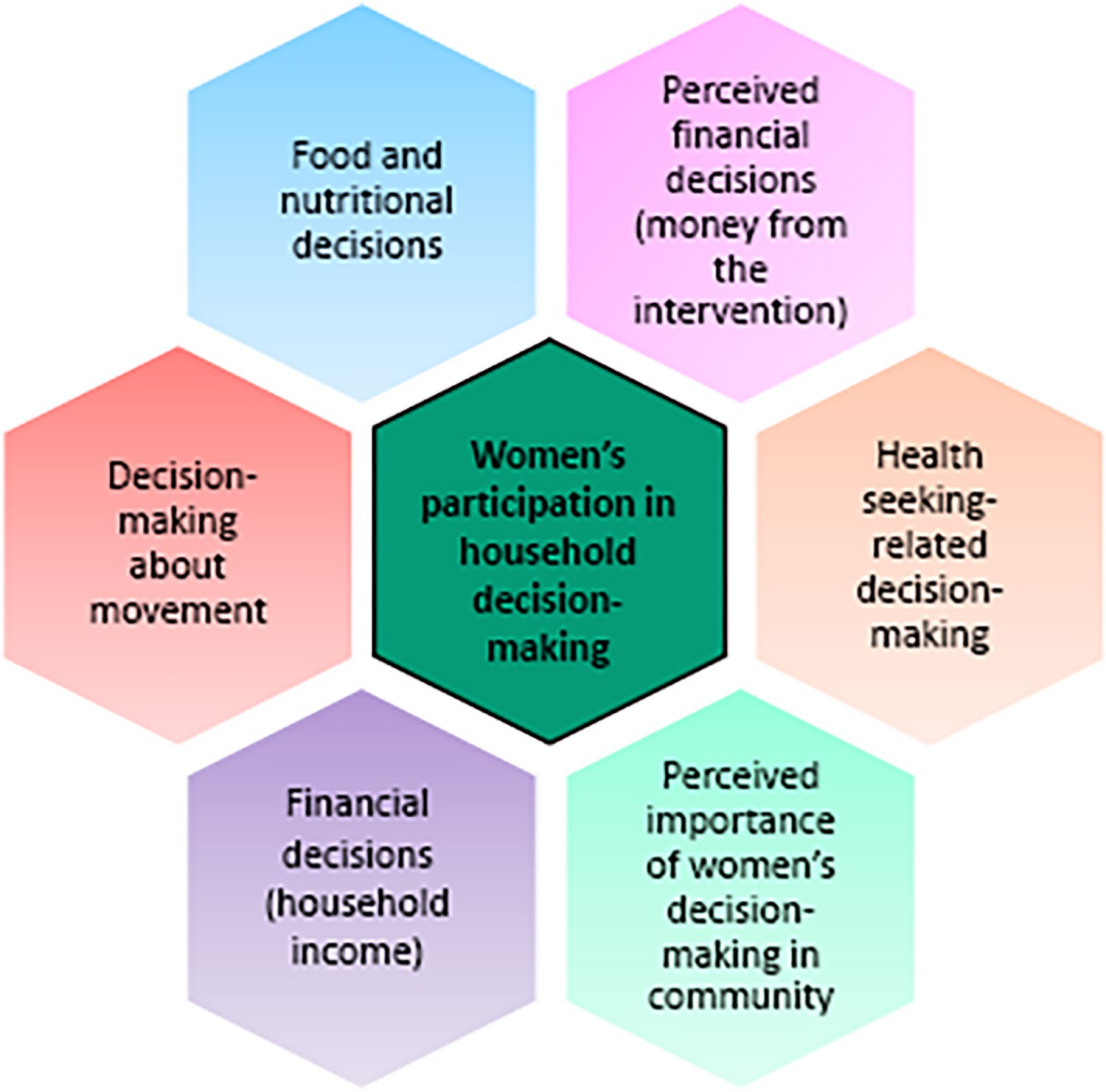
Areas of women’s participation in household decision-making.

In analysing the in-depth interview data, several key themes centred on decision-making emerged: women’s input into financial decisions, participation in health seeking-related decision-making; the overall importance attributed to women’s contribution to the decision-making process in the household and community; and decisions regarding freedom of movement. The areas of women’s participation in household decision-making are illustrated in Fig 1.

### Financial decision-making

#### Money from the intervention

When asked about the expenditure of the cash, most women described the need to inform or discuss the purpose of expenditure with their husbands or other family members. "*For buying small and tiny things*, *it is not necessary*. *However*, *for buying or spending for a big and expensive thing*, *it is mandatory to discuss with him (husband)"* (Pregnant woman, 25 years). When asked who would spend the cash and on what, one woman said her husband would use the money on food, "*He will buy food with the money which will heal my body*.*"* (Pregnant woman, 36 years). Women perceived that if they did not discuss the cash expenditure, they might face marital disharmony in their relationship.

Women noted that in very few instances, they would not need to explain to their husbands or family members, using the rationale that they directly received the cash. A few women stated that they would be free to spend the cash transfer as they wished without limitations. One woman who had not yet received the money said, *"I can spend as much as I want*. *If I spend this money*, *he will not say anything*. *If I don’t spend*, *he won’t say anything*.*"* (Pregnant women, 34 years). The women told of being free to spend the money as they liked, as their husbands know they do not spend money unnecessarily. However, one woman emphasised that it was more advantageous that she received the money as it would enable her to eat; *"It’s a little better if they give me money*. *I am a poor woman; I can eat if I have my own money"* (Pregnant women, 25 years).

#### Household income

Two of the women interviewed earned income; one ran a tailoring shop, and the other ran a poultry business from home, making four or five hundred Bangladeshi Takas per month (approximately 5 or 6 USD, average monthly wage 95–140 USD [51]). When asked whether they would spend this income on their own or in consultation with their husband, one reported handing over the income to her husband. The husband managed all the household income, and she informed us, "*I don’t have any other opinion*" (Pregnant woman, 36 years). Another woman reported needing to tell her husband about the purpose of expenditure for general household income.

*"He has trust in me that I will not do anything wrong*. *…with the money… (without informing him)*.*"* (Pregnant women, 34 years).

### Health seeking-related decision making

Women reported consulting with men when making health-related decisions. However, if a family member became sick, many women reported that their husbands would decide alone whether to take them to the doctor or hospital.

*"I had a stomach ache before*, *then I felt bad*, *I brought medicine*, *I didn’t feel well after eating*. *Then he said*, *’I will take you to the doctor*, *I will take you and see‴* (Pregnant woman, 36 years)

*"The decision would be made by my husband*. *As he is here*, *so he will make the decision"* (Pregnant woman, 24 years)

Whilst men may ultimately decide on the need for medical care; several women stated they had input into the decision-making process, discussing the problem together to make the best decision. However, when probed further, if the woman’s husband did not allow her to seek care, she would not disobey his directive.

*"We both have to make a decision*. *Q*: *Will he value your decision*? *A*: *Yes…*. *If he doesn’t let me go*, *I won’t go"* (Pregnant women, 25 years)

*"Decisions are taken by my husband and me–both of us*. *I suppose*, *he is the principal decision-maker*. *The main decisions are taken by my husband"* (Pregnant women, 32 years)

In some cases, older family members and relatives who were living close by were the key decision-makers regarding medical treatment. One woman reported that as her husband was away, working in Dhaka, her father-in-law would make the decision regarding medical treatment, and her mother-in-law would accompany her to the hospital if she required medical attention. Whilst another woman’s brother-in-law was living in an adjacent house, so the decision regarding medical treatment would be taken by him.

*"Decisions are taken by the guardian of the family*, *by my father-in-law*. *No matter whether he eats in this house or other house*. *He is the guardian of the family"* (Pregnant women, 20 years)

*"My younger sister-in-law’s husband takes decision while my husband is away*. *We called him and*, *as I am currently pregnant*, *I needed to tell him before going for any ANC check-up"* (Pregnant women, 32 years)

#### Food and nutritional decisions

In most households, women were the primary decision-makers regarding what foods would be cooked and eaten. Women cooked what they had directed their husbands to buy and what was seasonally available from the market, as they traditionally do not go to the marketplace. If a husband were away for work, the father-in-law would ask in advance what items such as onions, salt, cumin, and ginger were required, and he would then go to the market to purchase what was needed.

*"Women make most of the decisions when it comes to cooking*. *However*, *some men in the house do not understand*. *If at any time the food doesn’t taste good to them*, *they quarrel*. *Again*, *not all men in the house are the same*, *and not all women are the same …*.*"* (Pregnant woman, 36 years)

However, in other households, decisions as to what foods to purchase were decided by both women and men.

*"We both make decisions together*. *I told him again that there was no onion*, *no chili*, *he had to bring it; He brought it…*. *I asked him*, *"What will I cook*? *will I put potatoes in the curry*? *will I cook the fish*? *I ask him these…*. *Q*. *Both of you decided together*? *A*. *Yes"* (Pregnant women, 25 years)

All women reported deciding what to cook with the foods available, though this was mitigated by domestic workload and time factors.

*"Cooking items are adjusted according to work pressure*. *It can be seen that if the pressure is high*, *some light items (will be) cook(ed) on this day*, *and when the work pressure decreases*, *another item is cooked that day*. *Women make most of the decisions"* (Pregnant woman, 36 years)

#### Decisions on infant and young child feeding

Women spoke of understanding what foods their children liked and ate. When asked who decides what foods to cook for the baby or how much to feed the baby, all women responded that they alone made such decisions.

*"These decisions are mine*. *Because I am the mother of the child*. *Q*. *Do you make this decision alone*? *A*. *Yes*.*"* (Pregnant women, 32 years)

### Decision-making about movement

For some women, sociocultural norms restricted the freedom to move outside the home. Women needed to discuss their plans to go out with their husbands before going somewhere. One woman said she that in practice she could not go out without her husband’s permission; however, she liked to ask him and said that he also wants to be asked as he thinks that "wherever she goes, she tells me" (Pregnant women, 24 years). She said that her husband looked after her, offered to buy medicine for her when she was not well and encouraged her to go out and purchase medicine on her own.

*"I can’t go; I have to ask him… if there is necessary work*, *he will let me go and if there is no need*, *he does not permit it … When I told him to go to the clinic*, *he said*, *"You are unwell*, *you don’t need to go*, *I will buy medicine*.*" And buy it*. *This month I said*, *"I’ll go to buy medicine this time*.*" Then he said*, *"If you feel well*, *get courage then go*.*" Later I dared to go*.*"* (Pregnant woman, 36 years)

### Perceived importance of women’s decision-making in the community

We asked women how they thought other families in their local area valued the importance of women’s opinions when making decisions. Attitudes differed depending on the type of decision being made. Responses varied, with many women saying that they felt in three out of ten households in their local area, women had a voice and were listened to, leading to joint decision-making.

*"Men will do whatever they decide*. *Then listen to the women a little… many care about women’s words*. *Most husbands and wives will make decisions together*. *And if the man doesn’t*, *he decides alone"* (Pregnant women, 24 years)

However, nearly half of the women perceived that women had no voice in decision-making in seven out of ten households in their local area. When asked about assigning weight to a women’s opinion within a family, one woman responded, "Big or small whatever the decision it is taken solely by them (the male)" (Pregnant women, 25 years)

One woman’s attitude summed up the value and significance attributed to women’s input into decision-making by saying, "*A little less*, *isn’t it*? *Because they are ’girls’*, *so in any work*, *they are given little importance*" (Pregnant women, 25 years). Another woman responded when asked whether it was ok not to care about a women’s opinion or whether she would like to see a change in households that did not listen to women.

*"I want that*. *As a woman*, *if I don’t understand the pain of another woman*, *what else do I understand"* (Pregnant women, 32 years)

*"Those who are good consult their wives"* (Pregnant women, 34 years)

The overall opinion of women interviewed was that men in their community do realise that a woman’s input into decision-making is valuable. Yet ultimately, the man had the power to decide whether or not to listen and give weight to the woman’s opinion or make decisions on their own.

## Discussion

This study documents the current contextual information on the status of women’s participation in household decision-making at the start of the SCC Trial in rural Bangladesh. We explored perceptions, attitudes, or practices toward women’s participation in decision-making in order to qualitatively assess the SCC intervention’s impact on the women in the trial. Our findings detail the present state of power dynamics and relationships within the households to identify any disparities that occur along gender lines to enable us to assess any changes that may arise from the SCC Trial interventions.

Our study found that the majority of couples collectively determine how to allocate their finances. Ultimately, the husband or senior male family member determines health-related decisions. Women experienced more agency regarding decisions about the nutrition of their families as they alone usually decided on what to cook for their families and what to feed infants and young children. The status of women is a determinant of child nutrition outcomes, with studies from South Asia and Sub-Saharan Africa confirming that enhancing women’s status can have a powerful impact on child nutritional outcomes [52]. When a woman has agency, dietary quality, dietary diversity, and the intake of nutrients at a household level are enhanced [7].

Women in our study reported their husbands would be the ones to collect the cash from the bKash agent in the marketplace. In many parts of Bangladesh, it is socially unacceptable for women to go out due to purdah (female seclusion) and the restricted movement for women [53]. In contrast, a cash transfer programme in Nepal specified that women needed to collect the transfer personally each month; they could only receive the transfer at home if they were physically incapable of collecting it [54]. Yet another study in Nepal found that young married women could not go to the marketplace due to gender-based social norms and therefore had to rely on their husbands or mother-in-law to spend the cash [55]. In Kenya and Zimbabwe, women stated that they could not collect cash transfers if they could not produce a national identity card. While they could nominate people to collect the cash, the public perception was that a woman should collect the money [56]. Analysis of the unconditional cash transfer in Pakistan directed to women found that women in the programme experienced more freedom of movement, such as the ability to travel alone to relatives or see health care; however, these associations were not statistically significant [57].

Our research revealed that most women planned to jointly discuss the expenditure of the cash transfer and that their husbands would spend the money in the marketplace. Cash transfer programmes in Latin America have found that when women are the recipients of cash transfers and have a larger share of household income, they do not necessarily lead to a more significant say in household expenditure [13,57]. A review of The Government of Zambia’s unconditional cash transfer programme aimed at mothers with children under five living in poverty found that most household expenditure decisions were made by males, particularly if the decision was seen as important [58]. This finding differs from an unconditional cash transfer program that aimed to improve low birth weight in rural Nepal, which found women could independently decide how to spend the cash transfer yet not decide on other household income [55]. However, further analysis of this intervention revealed that women needed to spend the unconditional cash transfer as suggested; otherwise, they risked being chastised by their husbands, mothers-in-law, or community members [55]. The Livelihood Empowerment Against Poverty programme in Ghana is large-scale social protection targeted at those who live in extreme poverty [59]. Women in the programme would use the money to take care of their children and use it for household expenses without asking their husbands for permission or needing their husband’s money. In some cases, women even reported loaning money to their husbands. An unconditional cash transfer programme in Kenya that promotes women’s social and economic empowerment found that women were more likely to make decisions about household expenditure after receiving the cash transfer [60].

Our findings showed that whilst women had input into health care decisions, overall, men had the final say in decision-making. And when probed further, there was acceptance of this cultural norm to accept men’s role in decision-making, and women would not disobey their husband or senior male family member’s directive. A review of nationally representative surveys in South Asia revealed that in Nepal, India and Bangladesh, most women’s health care decisions were made without participation [61]. However, women receiving a transfer from Zambian Government’s Child Grant Programme indicated that they were in charge of making decisions regarding their health care [58].

Our findings reveal that women alone made decisions about what to cook for their families and directed their husband’s food purchases at the market. An unconditional cash transfer programme supporting poor mothers in Zambia reported similar findings as women said they were responsible and in charge of domestic decisions regarding food choices, household chores and children’s well-being [58]. This finding reflects women’s agency in making decisions around food as they alone made the decisions about infant and young child feeding. However, another study from India randomly allocating a cash transfer to a man or woman found no evidence for the cash transfer program’s influence on household consumption but did find evidence for programmatic impact on gender equalisation within household decision-making processes [62].

Evidence on the impact of cash transfers on a woman’s decision-making ability is varied, and programmes should not assume that if a woman receives a cash transfer, this will equate to changes in household decision-making ability. A review of a cash transfer programme in Egypt found that for women with no formal education, cash transfers reduced women’s control over decision-making [63].

Sociocultural factors influence decision-making, and many cash transfer programmes are assessed by asking recipients, mainly women, who make the decisions, with few studies interviewing both women and men within the household [57]. However, an analysis of cash transfer programmes in Kenya, Ghana, Zimbabwe and Lesotho revealed that social protection programmes could positively impact decision-making empowerment [64].

The SCC Trial enables women to access new resources such as the cash transfer, nutrition knowledge and mobile health technology. Studies assessing the impact of cash transfers from Sub-Saharan Africa and the Middle East revealed that the recipient’s confidence and psychosocial well-being improved in relation to others and at an individual level [64,65]. By monitoring studies and assessing if they improve the status and confidence of women, programs can augment nutrition outcomes whilst improving the status of women at the same time.

A strength of this study is that qualitative data will enable us to capture a deeper understanding into women’s participation in decision-making. This study has some limitations to consider when interpreting the findings. The lead author, EKK, does not speak Bangla; the transcriptions were checked randomly for quality and accuracy by Bangla speaking senior author (NAA). This study was conducted in a poor, rural community in Northern Bangladesh; therefore, these findings are not generalisable. However, our objective was not to generate generalisable findings but to shine light on the nuance of women’s experiences and perceptions around decision-making.

## Conclusions

This study documented women’s participation in decision-making at the start of the SCC Trial to provide critical information that will enable us to assess the impact of the trial. Baseline process evaluation data will provide valuable insight to help us assess changes in women’s agency and decision-making ability. Evidence has shown that interventions utilising cash transfers and behaviour change communication can potentially increase women’s autonomy and agency and alter gender based intrahousehold power dynamics; therefore, documenting and monitoring these effects to fill the evidence gap on impact pathways is essential.

## Supporting information

S1 Checklist Inclusivity in global research checklist. (DOCX)

S2 Checklist COREQ (COnsolidated criteria for REporting Qualitative research) checklist. (PDF)
